# Ravulizumab in myasthenic crisis: the first case report

**DOI:** 10.1007/s00415-024-12234-2

**Published:** 2024-02-23

**Authors:** Franz Felix Konen, Konstantin Fritz Jendretzky, Dominica Ratuszny, Ramona Schuppner, Kurt-Wolfram Sühs, Marc Pawlitzki, Tobias Ruck, Sven G. Meuth, Thomas Skripuletz

**Affiliations:** 1 Department of Neurology, Hannover Medical School, Carl-Neuberg-Str.1, 30625 Hannover, Germany; 2 Department of Neurology, Medical Faculty and University Hospital Duesseldorf, Heinrich Heine University, Moorenstraße 5, 40225 Duesseldorf, Germany

Dear Sirs,

Generalized myasthenia gravis (MG) is a chronic autoimmune disorder characterized by dysfunction of the neuromuscular junction due to antibodies that target this area [1]. The primary cause of postsynaptic damage is believed to be the activation of the immune and complement systems, a process initiated by the accumulation of antibodies [1]. The effects of this condition include weakened skeletal muscles and heightened fatigue, which can escalate to a myasthenic crisis. This crisis is characterized by an exacerbation of muscle weakness including bulbar muscles and the diaphragm, resulting in respiratory failure requiring invasive ventilation, and the necessity for intensive care [1]. Therapeutic approaches to a myasthenic crisis encompass the continuous administration of symptom-alleviating drugs, complemented by the initiation or enhancement of immunomodulatory therapies, including options like apheresis or intravenous immunoglobulins [1].

Ravulizumab, a humanized monoclonal antibody that specifically targets the terminal complement protein C5, has recently been approved for the treatment of generalized MG in patients testing positive for anti-acetylcholine receptor antibodies [2]. By incorporating four amino acid substitutions in comparison to the structure of eculizumab, ravulizumab achieves maintained therapeutic serum concentrations, allowing the therapy interval to be extended to 8 weeks [2, 3].

Here, we present a patient diagnosed with anti-acetylcholine receptor antibody-positive MG who experienced a refractory myasthenic crisis. This crisis was characterized by respiratory insufficiency and dysphagia, leading to the requirement for intensive care. Notably, there was an almost complete resolution of neuromuscular symptoms following the initiation of ravulizumab treatment.

A 34-year-old German woman with a history of generalized MG presented with worsening of neuromuscular symptoms including tetraparesis, head drooping, dysphagia, dysarthria, and intermittent dyspnea. Initially presenting with mild symptoms eight months prior, her MG diagnosis was confirmed four months earlier with anti-acetylcholine receptor antibodies (135 nmol/l; reference value 0.25) and response to acetylcholine esterase inhibitors. Her initial treatment included prednisolone (20 mg/day) and pyridostigmine (120 mg/day). Magnetic resonance imaging excluded thymoma and concurrent hypothyroidism was managed with levothyroxine.

At admission, the Quantitative Myasthenia Gravis (QMG) score was 18, the Myasthenia Gravis Activities of Daily Living (MG-ADL) score was 11 and the Myasthenia Gravis Foundation of America (MGFA) classification was IVa, thus infusion of intravenous immunoglobulins (IVIg) was promptly started, the oral dosage of pyridostigmine was increased (360 mg/day) and a high dosage prednisolone (80 mg/day) regimen was implemented. A summary of the treatment is illustrated in Fig. 1. The disease course presented as highly treatment refractory. Although the patient initially received treatment with IVIg (130 g over 5 days), her condition deteriorated, marked by respiratory fatigue, reduced forced vital capacity, evidence of non-compensated respiratory acidosis, subjective dyspnea with tachypnea, and the need for oxygen administration. This necessitated the transfer to intensive care, intravenous administration of pyridostigmine, and the implementation of immunoadsorption therapy (5 cycles), followed by another course of IVIg (50 g over 2 days). While there was an initial improvement, her symptoms again worsened, as indicated by varying QMG scores between 18 and 25, and MGFA classifications fluctuating between II and IV. Consequently, another course of immunoadsorption therapy (5 cycles) was required. Improvement in symptoms was achieved, and subsequently, rituximab (1000 mg) was administered as maintenance therapy. However, it failed to sustain the improved clinical condition, and the patient was still highly affected by myasthenic symptoms with a QMG score of 23 and a MGFA IVb. As a result, it was decided to initiate treatment with ravulizumab. About a month after the patient's first visit at hospital and a week after rituximab infusion, she received a 3000 mg infusion of ravulizumab, which was well-tolerated and did not cause any adverse events. 2 weeks later, the patient was in a condition to be discharged from the hospital. At this point, the QMG score was 6 and MGFA classification was IIa. The patient's ongoing medications included prednisolone (80 mg/day), pyridostigmine (450 mg/day), and amoxicillin. The latter was taken because no meningococcal vaccination could be carried out before the start of therapy with ravulizumab. Following discharge, the patient demonstrated no clinical worsening during the subsequent visits. This was evidenced by stable clinical scores and an observed enhancement in self-reported outcomes. Particularly noteworthy is the reduction in the MG-ADL score post-discharge, which fell from 4 during the initial evaluation (7 weeks post-first ravulizumab administration) to 2 at a subsequent assessment (15 weeks post-first administration of ravulizumab). Similarly, the Myasthenia Gravis Quality of Life (MG-QOL 15) score showed a significant improvement, dropping from 14 at the first visit after discharge to 7 at the second visit, reflecting an enhanced quality of life. By the time of the most recent visit, the dosage of prednisolone was successfully reduced to 20 mg/day (pyridostigmine dosage was still 450 mg/day). Up to the last presentation, after three cycles of ravulizumab, the patient reported no adverse events and showed no signs of clinical decline.

**Fig. 1:**
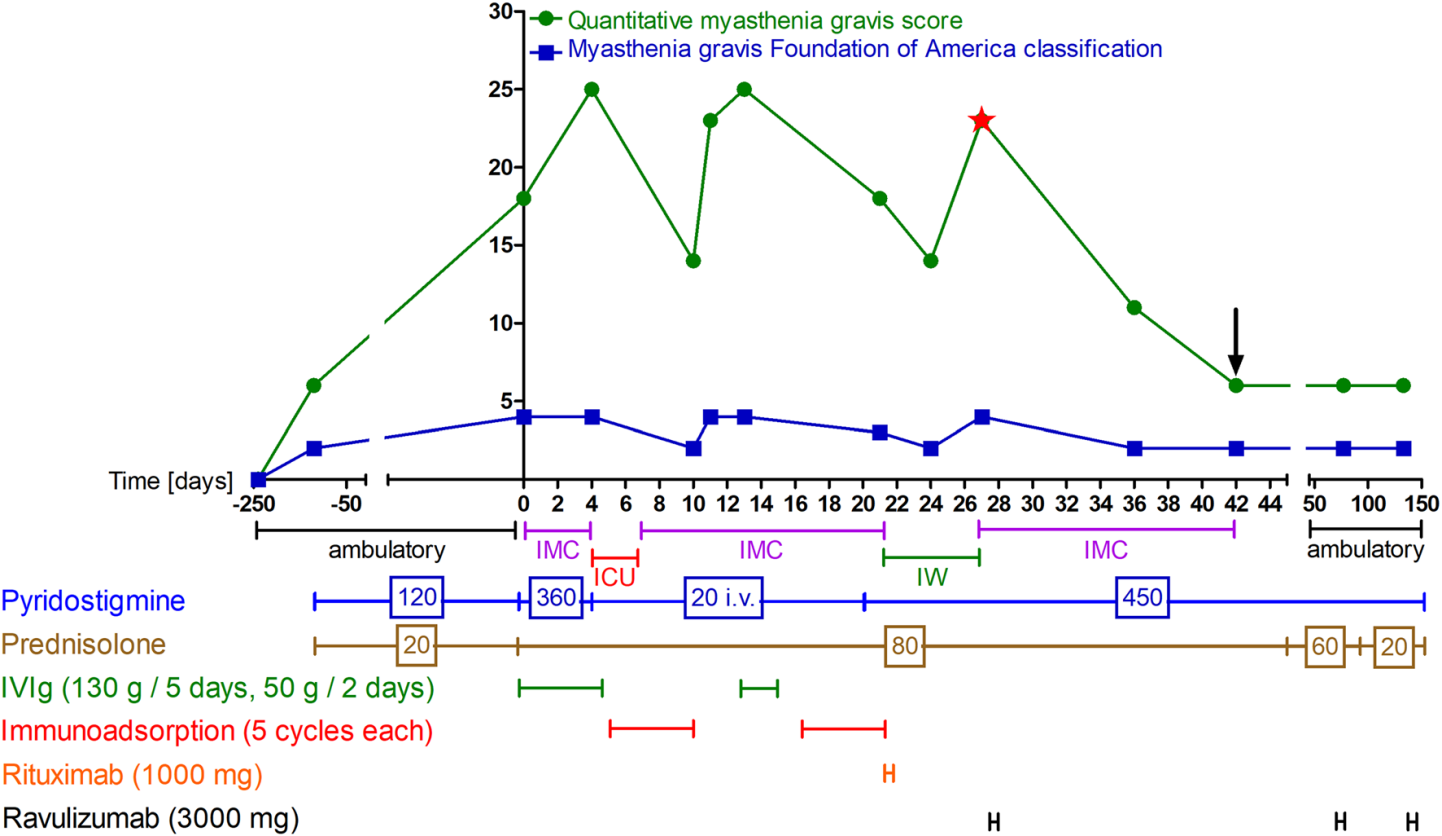
**Disease course.** *IMC* intermediate care unit, *ICU* intensive care unit, *IW* inpatient ward (no monitoring), *IVIg* intravenous immunoglobulins, *asterix* initiation of ravulizumab treatment, *arrow* discharge from hospital. The daily dosage in mg of pyridostigmine and prednisolone is given in the boxes of the referring therapies

In this case, we present the first use of ravulizumab in a patient with a severe myasthenic crisis who was resistant to multiple standard therapies, including IVIg, immunoadsorption, and rituximab. The administration of ravulizumab during the exacerbation phase led to significant improvement in neuromuscular functions such as swallowing, breathing, and muscle strength within a week. This improvement was sustained over a 19-week follow-up period, with further enhancements in MG-ADL and QOL observed after the second infusion of ravulizumab.

This case underlines the potential of complement inhibitors like ravulizumab as an additional treatment strategy for refractory myasthenic crises. The pathophysiology of myasthenic crisis, where complement-mediated damage is believed to play a crucial role, underscores the importance of using complement inhibitors in its treatment [4, 5]. Notably, similar rapid improvements were observed with eculizumab in four patients with myasthenic crisis, who had generalized, anti-acetylcholine receptor antibody-positive MG [6–10]. Complement inhibitors such as eculizumab and ravulizumab have shown to be fast-acting in clinical studies [6–13]. Moreover, eculizumab has demonstrated rapid positive effects within seven days in patients with Paroxysmal Nocturnal Hemoglobinuria (PNH) and Atypical Hemolytic Uremic Syndrome (A-HUS) [12, 13]. However, these agents are not yet approved for myasthenic crises.

A potential limitation to consider is that the observed durable stabilization as well as the long-term stability over 19 weeks might not be only attributed to ravulizumab initiation. Since it is known that rituximab develops its full therapeutic potential after 4–8 weeks, the combination of both mechanisms, CD20-depletion and complement inhibition, could be responsible for the long-lasting stability [14]. On one hand, this case demonstrates that a treatment regimen combining CD20-depletion, potentially more efficacious in early-stage disease, with ravulizumab, might significantly benefit patients with severe, newly diagnosed anti-acetylcholine receptor positive MG. This approach appears to hold promise for such patients. On the other hand, it is essential to recognize that the mechanism of action of ravulizumab could potentially influence the complement-mediated effects of rituximab. This potential interaction is an important consideration in the overall treatment strategy [3, 14]. However, this delayed onset of the therapeutic effect of rituximab once again underlines the rapid-acting mode of ravulizumab in the treatment of refractory myasthenic crisis [14]. Apart from rituximab, our patient was treated with multiple immunotherapeutics, indicating that these other immune therapies may also have contributed to the patient's condition. Consequently, it is possible that ravulizumab alone may not be solely responsible for the continued stabilization of the patient's health.

Given the patient's clinical stability on ravulizumab throughout the follow-up period, coupled with their satisfaction regarding the reduced frequency of visits to our facility, we plan to continue the ravulizumab treatment for our patient. Indeed, the initial rituximab treatment could have potentially led to sustained improvements. This suggests the opportunity to consider lowering the intensity of the ongoing ravulizumab therapy in the future. Such a decision will depend on the ongoing evaluation of the patient's condition and a deeper understanding of the long-term impacts associated with these therapies.

In conclusion, initiating complement inhibitors earlier during a myasthenic crisis might offer additional benefits. Randomized and controlled studies are essential to fully evaluate the effectiveness of complement inhibitors, including ravulizumab, in the management of myasthenic crises.

## Data Availability

Anonymized data not published within this article will be made available by reasonable request from any qualified investigator.
